# Letter: Time to correct the record on the global burden of myalgic encephalomyelitis/chronic fatigue syndrome (ME/CFS)

**DOI:** 10.1186/s12967-025-06281-0

**Published:** 2025-03-14

**Authors:** Maya Vardaman, Stuart Gilmour

**Affiliations:** Graduate School of Public Health, St. Luke’s International University, Tokyo, Japan

Dear Editor,

We have read Lim et al.’s study [1] with great interest. Studies of this illness are scarce, so their article is important in establishing the global prevalence systematically. In fact, it has been cited over 200 times and has had a significant influence on global reporting of the burden of ME/CFS. Unfortunately, the core figure that is most frequently drawn from the article is a significant underestimate of the prevalence of ME/CFS, and has led to the spread of misinformation about the burden of this disease.

A global ME/CFS population of 17 to 24 million people is reported in articles such as those published in *BMC Medicine* [2] and *Science* [3], all referencing Lim et al. The same range of numbers is also easy to find in patient advocacy websites and news reports, such as those of the American Myalgic Encephalomyelitis and Chronic Fatigue Syndrome Society [4] and CNN [5], all updated after Lim et al.’s article was published. However, this figure is drawn from the Background section of Lim et al.’s article, where they state, “In worldwide statistics, approximately 1% of the population, 17 to 24 million people, suffer from this condition [14], which is likely to be as common as rheumatoid arthritis” [1]. The cited reference (reference [14]) is an article about the global burden of multiple sclerosis, and is not relevant to the global ME/CFS population.

The actual headline result of the study by Lim et al. was an estimated prevalence of 0.89% using the CDC-1994 definition, as indicated in the Discussion and Conclusions sections of their article [1]. If we correctly apply this to the global population of 8 billion, the true prevalence of ME/CFS would be 71.2 million (71,200,000). However, new research using more up-to-date datasets and methods is essential to gain a full understanding of the burden of this disease.

Due to this unfortunate misreading and misattribution by others of an erroneous reference in the Background section of Lim et al.’s article, there is widespread underestimation of the true burden of ME/CFS. Such underestimation may exacerbate the isolation, stigmatization, and suffering of people living with this poorly-understood and under-recognized illness. More effort is needed to correct this underestimation globally, develop treatment methods and welfare support, and relieve the burden of ME/CFS. Correct reporting of the true prevalence of this disease is an important first step to addressing the ongoing neglect that its sufferers experience, and this can begin with the correct reporting of Lim et al.’s research.

## Data Availability

Not applicable.

## Abbreviations

ME/CFS: Myalgic encephalomyelitis/chronic fatigue syndrome
